# Stapled vs. hand-sewn anastomosis during esophagectomy: a randomized trials systematic review and meta-analysis

**DOI:** 10.1007/s13304-025-02464-y

**Published:** 2025-11-21

**Authors:** Matteo Calì, Alberto Aiolfi, Gianluca Bonitta, Michele Manara, Quan Wang, Antonio Biondi, Davide Bona, Luigi Bonavina, European Foregut Society (EFS) Collaborative Group, Yves Borbely, Moustafa Elshafei, Suzanne Gisberz, Christian Gutschow, Mark Ivo van Berge Henegouwen, Sheraz Markar, Calin Popa, Diana Schlanger, Sebastian Schoppmann, Aleksandar Simić, Ognjan Skrobic, Dimitrios Theodorou

**Affiliations:** 1 Division of General Surgery, Department of Biomedical Science for Health, I.R.C.C.S. Ospedale Galeazzi – Sant’Ambrogio, University of Milan, Via C. Belgioioso, 173, 20157 Milan, Italy; 2 Department of Biomedical Sciences for Health, Division of General and Foregut Surgery, University of Milan, IRCCS Policlinico San Donato, Milan, Italy; 3 Surgical Division, Department of General Surgery and Medical Surgical Specialties, G. Rodolico Hospital, University of Catania, 95131 Catania, Italy

**Keywords:** Esophageal cancer, Esophagectomy, Anastomotic leak, Anastomotic stenosis, Postoperative complications

## Abstract

**Introduction:**

Esophagogastric anastomosis during esophagectomy is a technically demanding step, carrying a high complication rate. Numerous techniques for anastomosis fashioning have been described, including hand-sewn (HS) and stapled (ST) anastomosis however, the optimal method remains uncertain.

**Purpose:**

Analyse short-term outcomes for ST vs. HS anastomosis.

**Methods:**

Systematic review and meta-analysis of randomized controlled trials (RCTs). PubMed, Scopus, Web of Science, Cochrane Central Library, and ClinicalTrials.gov were queried. Primary outcomes were anastomotic leak (AL) and stricture (AS).

**Results:**

Twelve RCTs (2015 patients) were included. All trials were deemed to have an intermediate risk of bias. ST anastomosis was performed in 51.9%. The age of the patient population ranged from 37 to 88 years and 73% were males. Squamous cell carcinoma was diagnosed in 76.9% of patients. Neoadjuvant therapy was completed in 32.9%. Ivor-Lewis or McKeown esophagectomy were performed with thoracic (57.2%) or cervical (42.8%) anastomosis. No significant differences were found for ST vs. HS anastomosis for AL (RR 0.97; 95% CI 0.70–1.35) and AS (RR 1.47; 95% CI 0.96–2.23). Further, no differences were found for cardiovascular complications (RR 1.09; *p* = 0.59), pulmonary complication (RR 1.12; *p* = 0.28), length of stay (SMD 0.03; *p* = 0.69), and 30-day mortality (RR 1.30; *p* = 0.18). Operative time was shorter in ST anastomosis (SMD − 0.11; *p* = 0.002).

**Conclusions:**

ST and HS esophagogastric anastomosis yield comparable rates of AL, AS, postoperative complications, and in-hospital mortality. The use of ST anastomosis may result in a shorter operative time. The choice of technique should be determined by the surgeon’s expertise and clinical scenario.

**Supplementary Information:**

The online version contains supplementary material available at 10.1007/s13304-025-02464-y.

## Introduction

Esophageal cancer ranks as the sixth most prevalent malignancy globally and is the eighth leading cause of cancer-related mortality. Prognosis remains poor, with five-year overall survival rates between 15% and 20% [1]. The current gold standard for treatment involves esophagectomy, lymphadenectomy, and the restoration of gastrointestinal continuity via gastric conduit reconstruction [2, 3]. Esophagogastric anastomosis is regarded as the most technically demanding step, carrying a high complication rate. Anastomotic leak (AL) occurs in up to 10% of patients and is associated with a threefold increase in mortality, extended hospitalization, delayed initiation of oral intake, risk of reintervention, increased recurrence rates, and reduced overall as well as disease-free survival [4,70]. Anastomotic stricture (AS) has been reported in up to 30% of cases [5, 6], often requiring endoscopic dilation and negatively affecting postoperative recovery, nutritional status, and quality of life [7].

Numerous techniques for esophagogastric anastomosis have been described, including hand-sewn (HS) and stapled (ST) anastomosis, each presenting distinct advantages and limitations in both thoracic and cervical settings [8]. The optimal method for esophageal anastomosis remains uncertain, as existing studies offer conflicting evidence and no definitive conclusions regarding superior outcomes leaving the choice of technique on the surgeon’s expertise and personal preference [9]. In recent decades, there has been a gradual shift from HS to ST techniques, driven by advancements in stapling technology and efforts toward standardizing practices [10]. Nevertheless, the recent introduction of robotic platforms—offering enhanced visualization, precision, and dexterity—has led to renewed interest in the HS anastomosis technique [11, 12].

This systematic review and meta-analysis seek to provide updated insights into the comparative short-term outcomes of ST versus HS anastomosis in patients undergoing esophagectomy for resectable esophageal cancer in the setting of randomized controlled trials (RCTs).

## Materials and methods

A systematic review was conducted in accordance with the Preferred Reporting Items for Systematic Reviews and Meta-Analyses (PRISMA) guidelines [13]. Literature searches were performed using PubMed, Scopus, Web of Science, Cochrane Central Library, Google scholar, and ClinicalTrials.gov, with the final search date set as 10 January 2025. The search terms included “Esophagectomy,” “Esophageal,” “Esophagus,” “Cancer,” “Hand-sewn,” “Stapler,” “Anastomosis,” “Anastomotic,” and “Leak”. These were used in combination with the Boolean operators AND or OR. Details of the comprehensive literature search are provided in Appendix 1. Relevant studies reporting short-term outcomes after esophageal resection for cancer and comparing HS vs. ST anastomoses, were selected. All titles were screened, relevant abstracts were reviewed, and reference lists of each article were independently assessed by three authors (AA, MC, and MM). The review protocol was registered with PROSPERO CRD420250654558.

### Eligibility criteria

Inclusion criteria: (a) RCTs evaluating short-term outcomes following esophagectomy for cancer in adult patients (≥ 18 years), comparing ST) versus HS anastomosis; (b) articles written in English; (c) when two or more papers were published by the same institution, study group, or used the same data-set, articles with the longest follow-up or the largest sample size; (d) in case of duplicate studies with accumulating numbers of patients, only the most complete reports were included for quantitative analysis. Exclusion criteria: (a) non-English written; (b) conference abstracts, review articles, and case reports; (c) studies that doesn’t report a comparison between previously mentioned anastomotic techniques; (d) non RCTs study.

## Data extraction

The following data were extracted: authors, year of publication, demographic characteristic of the included patients, tumor location, perioperative therapy, tumor histology, pathologic tumor stage, surgical radicality and surgical approach, operative time (minutes), hospital stay, short-term outcomes, and in-hospital mortality.

## Outcome of interest and definitions

The primary outcome assessed was AL. Secondary outcomes included AS, mortality, cardiopulmonary complications, hospital length of stay and operative time. AL was defined as evidence of contrast extravasation at postoperative swallow study and/or CT-scan, or endoscopic visualization of anastomotic dehiscence/fistula, or visible loss of digestive secretions through surgical drains combined with clinical signs. AS was defined based on the need for endoscopic dilatation or evidence of reduced anastomosis diameter at postoperative endoscopic or swallow studies assessment. Cardiovascular complication was defined as postoperative myocardial infarction, stroke, peripheral limb ischemia, thrombotic events, and pulmonary embolism. Pulmonary complication was defined as pneumonia, pleural effusion with lung atelectasis, acute respiratory distress syndrome (ARDS), and respiratory failure requiring reintubation. Esophageal cancer was defined as any primary histopathologically verified neoplasm situated in the cervical, thoracic, and distal esophagus or gastroesophageal junction (Siewert I–II).

## Quality assessment

Three authors (AA, MC, GB) independently assessed the methodological quality of the selected trials by using the Cochrane risk of bias tool II [14]. This tool evaluates five domains: (1) bias of randomization; (2) allocation concealment; (3) bias due to missing outcomes; (4) bias in the measurements of outcomes; and (5) bias in selection of the reported results. Thus, each RCT was graded as having low, moderate, or high risk of bias [15]. Disagreements were solved by discussion.

### Statistical analysis

The results of the systematic review were summarized quantitatively into frequentist random effect meta-analysis of pooled risk ratio (RR) and standardized mean difference (SMD). An inverse-variance method and DerSimonian–Laird estimator for the variance of the true effect size (τ^2^) were performed [16]. Heterogeneity among studies was evaluated by the I^2^ index and Cochran’s Q test. Statistical heterogeneity (I^2^) was stratified as low (< 25%), moderate (25–75%), or high and significant when *p* < 0.10 [17, 18]. The Wald-type 95% confidence interval (CI) was computed for pooled measurements; otherwise, the 95% CI for the I² index was calculated according to Higgins and Thompson [18]. The prediction interval for the treatment effect of a new study was calculated according to Borenstein et al. [17]. As the sample size was not the same in all studies, we performed a sensitivity analysis by excluding one study each time and rerunning the analysis to verify the robustness of the overall results. The publication bias was also investigated with the trim and fill funnel plot and Egger test. A two-sided p value was considered statistically significant when *p* < 0.05. All analyses and figures were carried out using the R software program, version 3.2.2 [19].

## Results

### Literature search

**Fig. 1:**
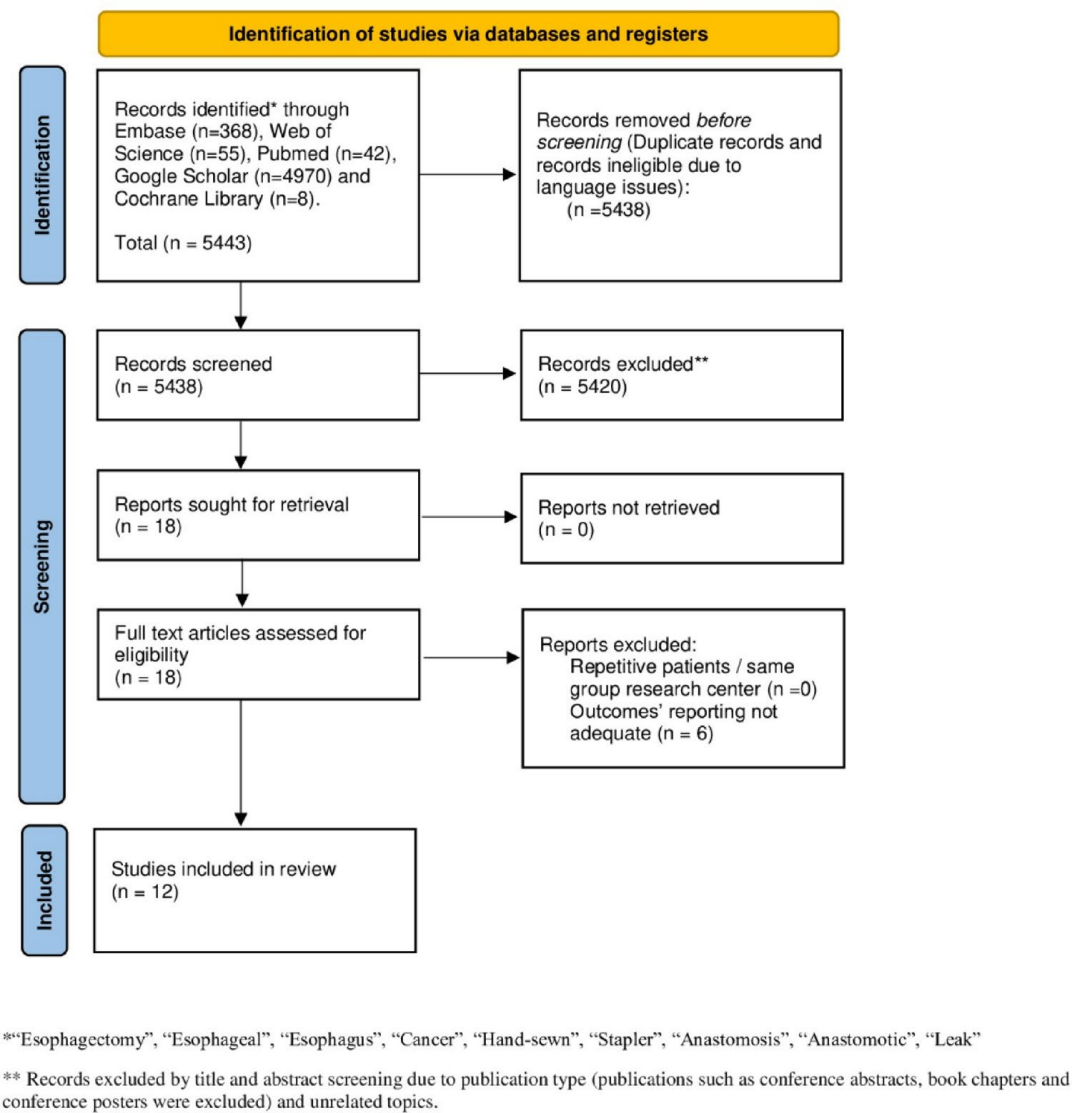
The Preferred Reporting Items for Systematic Reviews and Meta-Analyses (PRISMA) diagram

The PRISMA flow chart is reported in Fig. 1. Overall, 5443 publications were identified. After duplicates removal, 5438 titles were screened. After title and abstract evaluation 18 articles were found possibly relevant for full-text assessment. After full-text analysis, 12 [20–31] studies meet the inclusion/exclusion criteria and were encompassed in the quantitative synthesis.

## Risk of bias assessment

The quality of included RCTs is reported in Supplementary Table 1. All RCTs had a single-center design. Randomization methods were specified in nine RCTs [20–22, 24, 25, 27, 28, 30, 31], while details on the blinding of perioperative outcomes were provided in one study [31]. Power analysis was clarified in five RCTs [20, 28–31]. Three RCTs provide specific data regarding the operating surgeon proficiency [20, 23, 31] whereas none reported specific details regarding the surgical quality control. Given all these limitations regarding randomization, power analysis, blinding, outcomes assessment and lack of quality controls all the included trials were deemed to have an intermediate risk of bias (Supplementary Fig. 1).

### Systematic review

Overall, 2015 patients that underwent esophagectomy for cancer were included (Table 1 [20–31]). Of those 1045 (51.9%) underwent ST while 970 (48.1%) underwent HS anastomosis. The age of the patient population ranged from 37 to 88 years, the majority (72.7%) were males. The preoperative BMI was not reported in any of the included trials and the ASA score was reported in one study [31]. Tumor histology was specified in 6 studies [21, 23–25, 29, 31]; squamous cell carcinoma and adenocarcinoma were diagnosed in 76.9% and 20.7% of patients, respectively. Pathological tumor staging according to the 7th edition of the American Joint Committee on Cancer (AJCC) and 6th Union for Internation Cancer control (UICC) was detailed in 8 studies [21, 24–26, 29–31] (stage 0–I: 16.8%, stage II: 39.7%, stage III: 43.2%, and stage IV: 0.3%). The tumor was located in the upper (5.6%), middle (57.5%), and lower (36.9%) esophagus. Neoadjuvant chemotherapy or chemoradiation therapy was defined in 5 studies [23, 24, 28, 30, 31] (891 patients) and completed in 32.9% of patients with heterogeneous strategies (i.e. protocols, regimens, dosages, radiation fractioning, etc.). Open Ivor-Lewis or McKeown esophagectomy were performed depending on operating surgeon preference, tumor location, and histology, all procedures and anastomoses were performed with open technique. Cervical and intrathoracic anastomosis were fashioned in 42.8% and 57.2% of patients, respectively. The technique for anastomosis was detailed in all studies; HS anastomosis was performed with one layer in six studies and two layers in five studies with both absorbable and non-absorbable sutures as per operating surgeon preference. Circular stapled end-to-end or end-to-side anastomosis with diverse stapling devices and size was described in 10 studies [20–27, 29, 30]. Linear stapled anastomosis was labeled in 2 studies [28, 31] both performing cervical Orringer or Collard side-to-side anastomosis (Supplementary Table 2).

**Table 1:** Demographic and clinical characteristics of patients undergoing hand-sewn (HS) or stapled (ST) anastomosis. Tumour location (T Location); neoadjuvant therapy (NADJ); histology (Squamous cell carcinoma SCC – Adenocarcinoma ADK – Other); hospital length of stay (HLOS), cardiovascular complication (CV), pulmonary complication (P). Data are reported as numbers, mean ± standard deviation, median (range)

Author, country, year	Period	Anastomotic technique	No. pts	Age (yrs)	M/F	Tumor location (U-M-L)	NADJ	Histology SCC-ADK-Other	Stage 0-I	Stage IIa	Stage IIb	Stage III	Stage IV
Valverde, France, 1996 ^20^	1991–1994	HS	74	59 ± 9	67/7	0-39-35	nr	nr	nr	nr	nr	nr	nr
		SA	78	59 ± 10	71/7	0-42-36	nr	nr	nr	nr	nr	nr	nr
Law, Hong Kong, 1997 ^21^	1989–1995	HS	61	64 ± 1,2	54/7	0-50-11	nr	61-0-0	3	9	5	44	0
		SA	61	63 ± 1	53/8	0-51-10	nr	61-0-0	2	6	2	51	0
Laterza, Italy, 1999 ^22^	1993–1996	HS	21	50,9	4/17	6-12-3	nr	nr	nr	nr	nr	nr	nr
		SA	20	51,9	3/17	4-12-4	nr	nr	nr	nr	nr	nr	nr
Walther, Sweden, 2003 ^23^	1990–1996	HS	41	68 ± 8,25	28/13	3-20-16	0	25-14-2	7	5		27	0
		SA	42	66 ± 10	29/13	1-10-24	0	17-18-7	6	6		23	0
Hsu, Taiwan, 2004 ^24^	1996–1999	HS	32	63 ± 10	27/5	10-10-12	17	32-0-0	2	6	7	17	0
		SA	31	61 ± 12	30/1	6-16-9	16	31-0-0	1	5	7	18	0
Okuyama, Japan, 2007 ^25^	1996–1999	HS	18	64,3 ± 6,75	16/2	0-13-5	nr	17-0-1	6	1	7	4	0
		SA	14	63,6 ± 3,75	13/1	0-10-4	nr	13-0-1	4	1	2	7	0
Luechakiettisak, Thailand, 2008 ^26^	2000–2005	HS	59	63.6 ± 7,25	50/9	0-31-28	nr	nr	1	12		46	0
		SA	58	62 ± 7,25	48/10	0-26-32	nr	nr	2	14		42	0
Zhang, China, 2010 ^27^	2002–2007	HS	244	60 ± 1.3	142/102	125 *- 119 **	nr	nr	nr	nr	nr	nr	nr
		SA	272	59 ± 1.2	158/114	134 *- 138 **	nr	nr	nr	nr	nr	nr	nr
Saluja, India, 2012 ^28^	2004–2010	HS	87	50.9 ± 14	54/33	0-45-35	51	nr	nr	nr	nr	nr	nr
		SA	87	51.4 ± 12	61/26	0-39-45	56	nr	nr	nr	nr	nr	nr
Wang, China, 2013 ^29^	2007–2008	HS	52	58.9 ± 7.3	40/12	0-43-9	nr	131 -12-1	6	19	3	22	2
		SA	92	61.4 ±7.7	82/10	0-65-27	nr		10	28	6	48	0
Liu, China, 2015 ^30^	2009–2011	HS	237	61 ± 9	176/61	42-138-57	29	nr	26	65	80	66	nr
		SA	241	62 ± 8	183/58	40-145-56	35	nr	28	70	79	64	nr
Nederlof, Netherlands, 2020 ^31^	2011–2014	HS	44	65 ± 23	40/4	34 * − 10 **	39	7-36-1	23	3	6	12	0
		SA	49	64 ± 19	36/13	38 * − 11 **	46	18-31-0	20	6	5	16	2

### Meta-analysis – primary outcomes

**Fig. 2:**
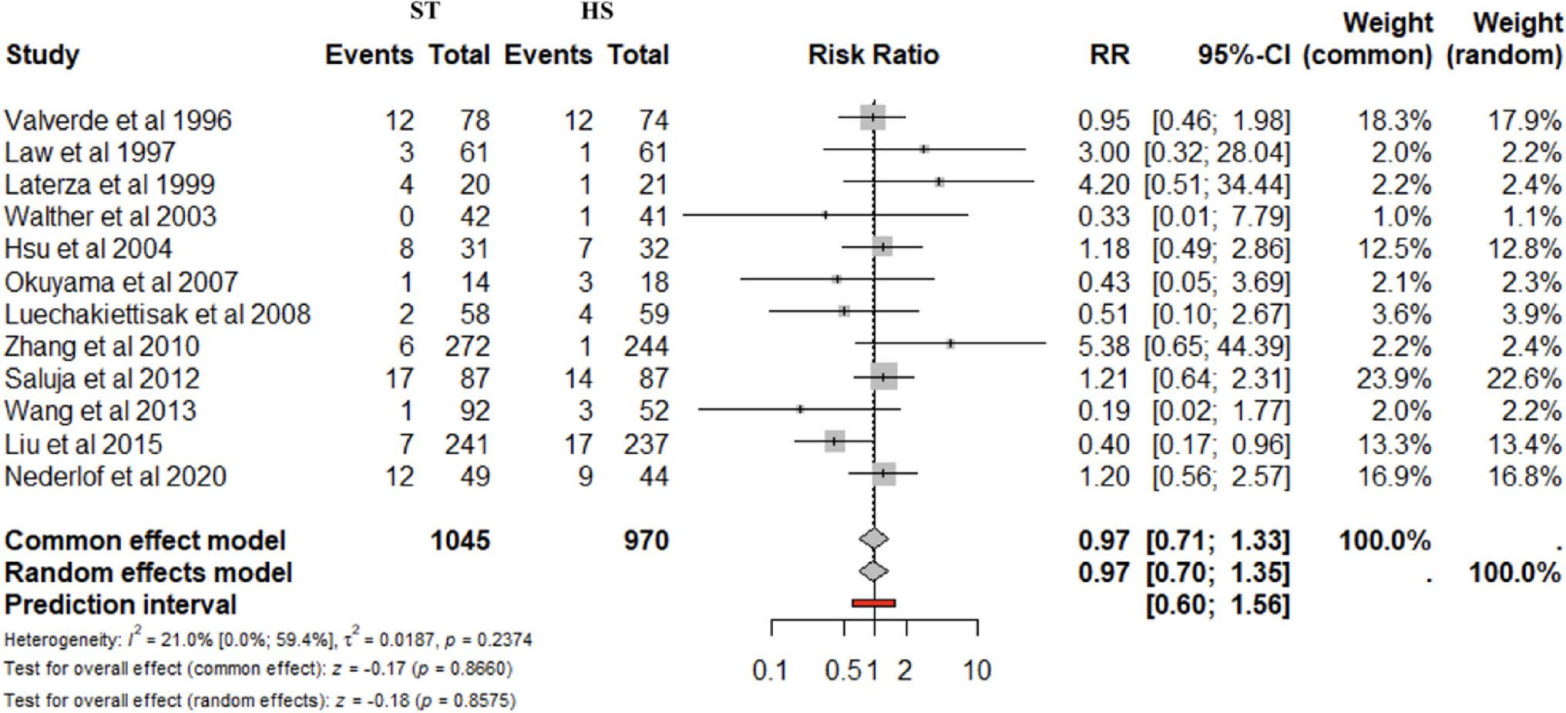

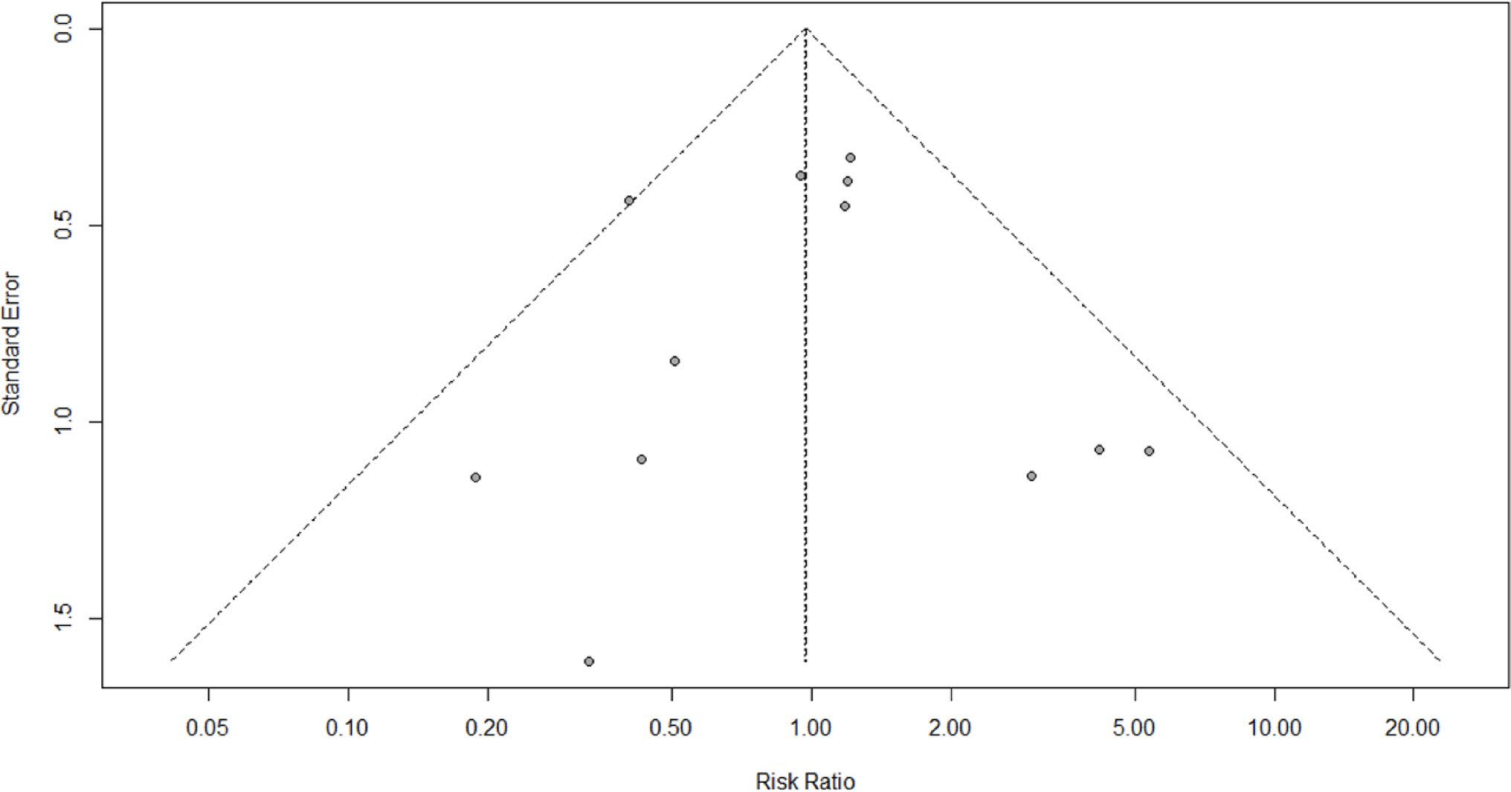
Anastomotic leak. Forrest **A** and Funnel **B** plot. RR: Risk ratio; 95% CI: Confidence Interval

AL was reported in all studies (2015 patients). The cumulative incidence of AL was similar between ST and HS anastomosis (6.9% vs. 7.5%). No significant differences were found for ST vs. HS (RR 0.97; 95% CI 0.70–1.35; *p* = 0.857) (Fig. 2A). The prediction lower and upper limits were 0.60 and 1.56, respectively. The heterogeneity was 21% (I^2^ = 21%) and τ^2^ = 0.01. The funnel plot (Fig. 2B) and the Egger test (*p* = 0.95) did not show evidence of publication bias. The sensitivity analysis showed the robustness of these findings in terms of point estimation, relative confidence intervals, and heterogeneity. No differences were found for ST vs. HS after stratification for cervical (4 studies) (RR 1.30; 95% CI 0.87–1.92; *p* = 0.727; I^2^ = 0.0%) and thoracic anastomosis (3 studies) (RR 1.76; 95% CI 0.39–7.99; *p* = 0.182 I^2^ = 41.2%).

**Fig. 3:**
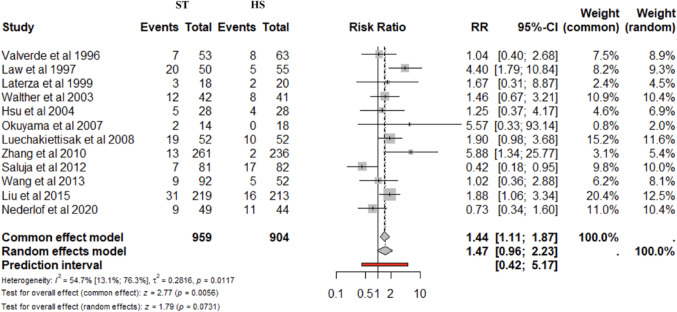

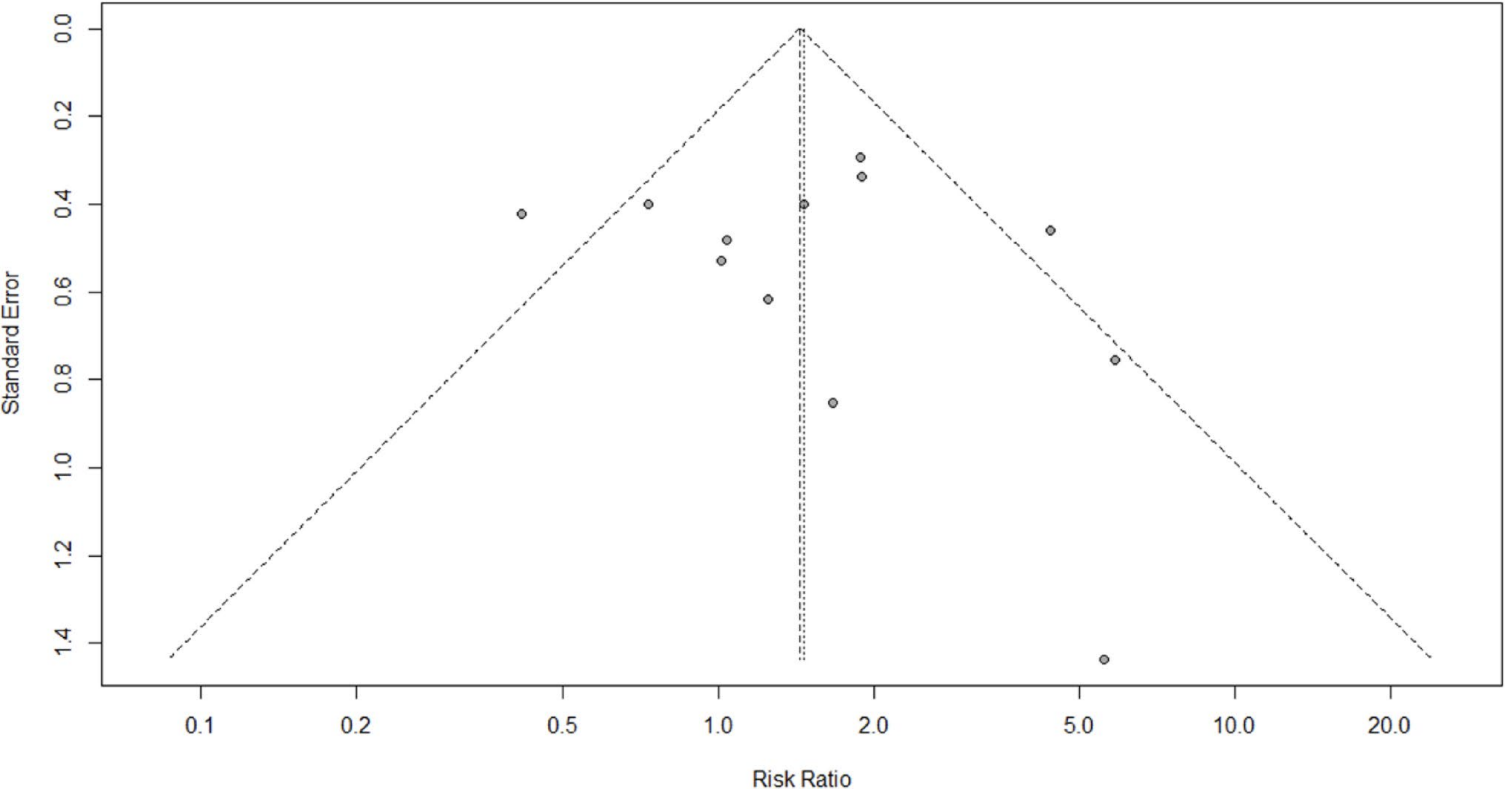
Anastomotic stricture. Forrest **A** and Funnel **B** plot. RR: Risk ratio; 95% CI: Confidence Interval

AS was reported in all studies and for 1863 patients (considering lost at follow-up). The cumulative incidence of AS was similar between HS and ST anastomosis (4.7% vs. 7.3%). No significant differences were found for ST vs. HS (RR 1.47; 95% CI 0.96–2.23; *p* = 0.073) (Fig. 3A). The prediction lower and upper limits were 0.42 and 5.17, respectively. The heterogeneity was moderate (I^2^ = 54.7%) and τ^2^ = 0.28. The funnel plot (Fig. 3B) and Egger’s test (*p* = 0.04) suggest potential asymmetry, which may indicate the presence of publication bias. Nevertheless, sensitivity analyses confirmed the robustness of the findings in terms of point estimates, confidence intervals, and heterogeneity. No differences were found for ST vs. HS after stratification for cervical (4 studies) (RR 1.53; 95% CI 0.75–3.11; *p* = 0.451; I^2^ = 72.2%) and thoracic anastomosis (3 studies) (RR 1.61; 95% CI 0.49–4.27; *p* = 0.197 I^2^ = 38.4%).

### Meta-analysis – secondary outcomes

No significant differences were found for ST vs. HS anastomosis when considering cardiovascular complications (RR 1.09; *p* = 0.59), pulmonary complication (RR 1.12; *p* = 0.28), HLOS (SMD 0.03; *p* = 0.69), and 30-day mortality (RR 1.30; *p* = 0.18) (Table 2). OT was significantly shorted in ST vs. HS anastomosis (SMD − 0.11; 95% CI -0.21, -0.03; *p* = 0.002).

**Table 2:** Summary of the analysis of the categorical and continuous outcomes comparing HS vs. ST

Outcomes	No. Studies	No. pts		Heterogeneity	
				I^2^ (%)	95% CI
Mortality	11	1871	1.30 (0.88; 1.93) ^♦^	0	0–60
Cardiovascular complications	7	1502	1.09 (0.80; 1.49) ^♦^	0	0–71
Pulmonary complications	9	1656	1.12 (0.91; 1.37) ^♦^	0	0–65
Operative time (min)	10	1890	-0.11 (-0.21; -0.03) ^*^	0	0–62
HLOS (day)	5	891	0.03 (-0.10; 0.16) ^*^	0	0–79

♦ Risk ratio, * Standardized mean difference, (95% Confidence Interval), I^2^ Heterogeneity

## Discussion

This meta-analysis indicates that ST and HS esophagogastric anastomosis yield comparable rates of AL, AS, postoperative complications, and in-hospital mortality. The use of ST anastomosis may result in a shorter operative time.

Esophagogastric anastomosis is regarded as the most technically demanding step of esophagectomy [32]. AL is a major complication, with reported incidence rates between 10% and 20% [1]. This complication is associated with increased postoperative morbidity, elevated mortality rates, prolonged hospitalizations, and higher healthcare costs [4, 5, 33, 34]. Moreover, AL has been shown to negatively affect long-term quality of life and oncological outcomes [4, 7]. Several factors may contribute to anastomotic failure, including the absence of a serosal layer and longitudinal orientation of muscle fibers, tension at the anastomotic site, anatomical location (cervical vs. thoracic), insufficient blood supply to the gastric conduit, surgical approach, malnutrition, and patient comorbidities [35–37]. Additionally, emerging evidence suggests that the gut microbiome may influence suture line healing and potentially contribute to anastomotic breakdown [38]. The specific technique employed for esophagogastric anastomosis has also been examined in prior studies as a variable that may affect leakage and stenosis [39]. ST and HS anastomosis are widely utilized globally, with selection often guided by the operating surgeon’s preference. However, despite a recent gradual shift toward ST anastomosis, definitive evidence favoring one technique over the other remains to be established. In our study, the estimated incidence of AL for HS and ST anastomosis was 7.5% and 6.9%, respectively. No significant differences were found in the quantitative analysis (RR 0.97) with a low related heterogeneity (I^2^ = 21%). Our findings are similar to what previously reported by Markar et al. [40] who concluded similar postoperative odds for AL comparing HS vs. ST (OR 1.06, 95% CI = 0.62–1.80, *p* = 0.83). Similar results were found by Honda et al. [41] that concluded equivalent AL rates for ST vs. HS anastomosis (RR 1.02, 95% CI: 0.66–1.59). In contrast Järvinen et al. [42] concluded higher leak rates for HS compared to ST anastomosis (OR 2.02; 95% CI 1.48–2.75). Since our findings indicate no significant difference in postoperative AL rates between HS and ST anastomosis techniques, we can argue that the choice of technique to fashion the anastomosis has minimal effect on the risk of postoperative AL.

In current literature, AS has been documented in up to 30% of cases, frequently necessitating endoscopic dilation. This complication may adversely impact postoperative recovery, nutritional status, and overall quality of life. In the present study, we observed a comparable postoperative AS risk between ST and HS anastomoses (RR 1.47). The heterogeneity among studies was moderate (I²=54%) however, the sensitivity analysis confirmed the robustness of the point estimate and 95% confidence intervals. Notably, the point estimate was above 1 thus suggesting a potential clinical trend toward an increased risk of AS following ST as compared to HS anastomoses. This effect may be influenced by the prevalent use of circular stapled anastomoses rather than linear stapled techniques in the included RCTs. According to previous studies, the use of circular staplers for esophagogastric anastomosis might be associated with an increased risk of postoperative stenosis compared to linear and HS anastomosis [43]. This effect is attributed to a reduced anastomotic diameter, greater incidence of staple line scarring/fibrosis, and a lack of mucosa-to-mucosa apposition. Further, the circular stapled technique produces an inverted anastomosis in which the esophageal and gastric mucosal margins are separated by muscle layers, potentially resulting in higher rates of stricture formation. Our findings seem to align with those reported by Markar et al. [40] (OR 1.76; 95% CI, 1.09–2.86; *p* = 0.02) and Honda et al. [41] (RR 1.67; 95% CI 1.16–2.42), both demonstrating a higher risk of AS following ST in comparison to HS anastomosis. Although heterogeneity was low to moderate, our findings should be interpreted with caution due to potential confounding factors. These include tensions-free visceral approximation with visceral size matching, adequate blood supply at the apex of the gastric conduit [44], patient age and comorbidities, steroid administration, body morphometrics with poor nutritional status, medical history (i.e. diabetes, peripheral vasculopathy), intraoperative variables (i.e. blood transfusion, intraoperative hypotension), variations in anesthesia protocols, epidural catheter utilization, neoadjuvant therapy, and tumor characteristics (i.e. R0). Further, it is important to carefully assess the absence of standardized anastomotic techniques, as inter-operator differences exist (e.g., single-layer versus dual-layer), along with variability in circular stapler sizes (21 mm, 25 mm, 28 mm, and 31 mm), types of stapling devices (such as purse-string suture, EEATM, OrvilTM), and incidence of postoperative leaks [45, 46]. The anastomosis location is a significant factor influencing the incidence of postoperative AL and AS, with cervical anastomoses demonstrating a higher susceptibility to such complications [47–51]. Importantly, subgroup analysis based on anastomosis site (cervical versus thoracic) yielded comparable outcomes. The selection of cervical versus thoracic anastomosis in the referenced studies was not randomized but determined by tumor characteristics/location, and surgeon preference with a risk for selection bias. Notably, other than oncological considerations, some technical issue should be considered when choosing the anastomotic technique in relation to the anatomical location and some clinical considerations [52]. For instance, circular staplers are advantageous for constructing anastomoses at the apex of the thorax (*cupula pleuralis*) however the anastomotic lumen is dependent on the original esophageal diameter with problems related to possible size mismatch [53]. Conversely, HS and linear stapled anastomoses generate larger luminal diameters thus minimizing possible issues related to visceral mismatch. However, these techniques necessitate a longer esophageal remnant, posing challenges to the creation of a well-aligned, high intra-thoracic tension-free anastomosis due to spatial constraints. The choice of surgical approach can impact the anastomotic technique. Evidence indicates that both circular and linear stapled anastomoses are generally preferred for transthoracic minimally invasive esophagogastric procedures due to the complexity of suturing within a rigid anatomical compartment using non-articulated instruments [43, 54]. Conversely, the robotic transthoracic approach offers enhanced surgical stability, allowing for greater precision and instrument dexterity, which facilitates direct suturing [55, 56].

No differences were found in term of postoperative cardiovascular and pulmonary complications with equivalent hospital length of stay and in-hospital mortality rates. However, we observed a significantly shorter OT for ST compared to HS anastomosis (SMD − 0.11; *p* = 0.002). This finding might reflect the reduced time to complete the anastomosis in a stapled fashion as described by Walther [23] and colleagues that concluded a significantly longer time to perform a manual single-layer compared to a stapled anastomosis (28 vs. 15 min; *p* < 0.001). Also Saluja et al. [28] concluded a significantly reduce time to complete a stapled compared to a double-layer manual anastomosis (27 vs. 25 min; *p* = 0.02). Our findings are in accordance with Honda et al. [41], who reported a notably reduced OT for ST versus HS anastomosis (mean difference: − 15.3 min; range: − 28.1 to − 2.39). Also Markar et al. concluded significantly reduced OT for ST vs. HS anastomosis (weighted mean difference − 1.56; *p* = 0.04). The decreased operative time likely reflects less tissue manipulation and a more standardized approach associated with ST anastomosis compared to the HS technique.

It is important to recognize that both AL and AS are influenced not only by the anastomotic technique itself but also by factors such as surgeon proficiency, the learning curve, structured training or mentorship programs, and hospital case volumes [57–59]. Achieving optimal surgical outcomes following esophagectomy depends significantly on surgical volume and the expertise of the operating surgeon. Evidence indicates that centralizing cases in high-volume centers contributes to lower mortality rates and may enhance overall outcomes [60, 61]. Notably, studies have shown that during the learning curve, AL rates decrease from 18% in the initial phase to 4.5% after 119 cases [62]. These observations underscore that AL and AS metrics do not exclusively reflect technical choices but are substantially affected by the learning curve and the surgeon’s experience. Furthermore, advancements such as fluorescence imaging with indocyanine green for gastric conduit perfusion assessment [63–65] and the implementation of robot-assisted esophagectomy [66, 67] could provide additional improvements in patient outcomes.

The study was conducted in accordance with PRISMA guidelines, following the comprehensive methodology established by the PROSPERO protocol. This process included thorough outcome measures and quality assessments at the study level, specifically addressing risk of bias for each included RTC. The selection criteria led to a relatively homogenous population for primary outcomes, as indicated by low to moderate heterogeneity. Nonetheless, certain limitations warrant consideration. First, variations in surgeon proficiency may influence postoperative complication rates. Second, potential confounders—such as inconsistencies in surgical approaches (e.g., Ivor Lewis versus McKeown), anastomosis location (cervical or intrathoracic), and standardized postoperative management protocols—could impact some of the assessed outcomes. Importantly, as all included trials reported outcomes exclusively for open esophagectomy, our findings should be interpreted with caution in the context of minimally invasive settings. Third, substantial technical variability existed in anastomotic techniques due to individual surgeon preferences. Fourth, differences in the definitions and reporting of outcomes among the included studies were observed and might introduce publication bias. Fifth, the effects of neoadjuvant therapies on short-term postoperative outcomes remain uncertain and require further investigation [68, 69]. Furthermore, there is a potential for temporal bias since the included studies encompass more than thirty years, which may result in heterogeneity related to chemoradiation protocols as well as intraoperative and postoperative management approaches. Lastly, because the majority of studies were conducted in Eastern countries, the applicability of these findings to other populations may be limited.

## Conclusions

This meta-analysis indicates that ST and HS esophagogastric anastomosis yield comparable rates of AL, AS, postoperative complications, and in-hospital mortality. The use of ST anastomosis may result in a shorter operative time. Ultimately, the choice of technique should be determined by the surgeon’s expertise and clinical scenario, as both ST and HS anastomosis demonstrate safety and efficacy when performed appropriately.

## Supplementary Information

Below is the link to the electronic supplementary material.

Supplementary Material 1

Supplementary Material 2
