# Supplementary material for: Stapled vs. hand-sewn anastomosis during esophagectomy: a randomized trials systematic review and meta-analysis
Source: Updates Surg. 2025 Nov 21;78(1):95–106. doi: 10.1007/s13304-025-02464-y (PMC12909439; doi:10.1007/s13304-025-02464-y)
Supplement: Supplementary file 1 — Supplementary Material 1 [file 13304_2025_2464_MOESM1_ESM.docx]

| **Author, year, country** | **Study design** | **Method of randomization** | **Surgeons' eligibility** | **Surgical quality control** | **Blinding** | **Power analysis** |
| --- | --- | --- | --- | --- | --- | --- |
| Valverde, France, 1996 ^20^ | nr | Blind enveloppe method  Stratified | >5y experience in esophageal surgery  >30 anastomoses/year per surgeon | nr | nr | Y |
|  |  |  |  |  |  |  |
| Law, Hong Kong, 1997 ^21^ | nr | Blind enveloppe method | nr | nr | nr | nr |
|  |  |  |  |  |  |  |
| Laterza, Italy, 1999 ^22^ | nr | Blind enveloppe method | nr | nr | nr | nr |
|  |  |  |  |  |  |  |
| Walther, Sweden, 2003 ^23^ | nr | nr | single experienced surgeon | nr | nr | nr |
|  |  |  |  |  |  |  |
| Hsu, Taiwan, 2004 ^24^ | nr | Blind enveloppe method | nr | nr | nr | nr |
|  |  |  |  |  |  |  |
| Okuyama, Japan, 2007 ^25^ | nr | Blind enveloppe method | nr | nr | nr | nr |
|  |  |  |  |  |  |  |
| Luechakiettisak, Thailand, 2008 ^26^ | nr | nr | nr | nr | nr | nr |
|  |  |  |  |  |  |  |
| Zhang, China, 2010 ^27^ | nr | According to clinical chart number | nr | nr | nr | nr |
|  |  |  |  |  |  |  |
| Saluja, India, 2012 ^28^ | nr | Computer-based non-stratified 1:1 randomization | nr | nr | nr | Y |
|  |  |  |  |  |  |  |
| Wang, China, 2013 ^29^ | nr | nr | nr | nr | nr | Y |
|  |  |  |  |  |  |  |
| Liu, China, 2015 ^30^ | nr | According to clinical chart number  Stratified | nr | nr | nr | Y |
|  |  |  |  |  |  |  |
| Nederlof, Netherlands, 2020 ^31^ | nr | Computer based hidden block size of 10  Stratified | 3 experienced surgeons | nr | Single-blinded | Y |
|  |  |  |  |  |  |  |
|  |  |  |  |  |  |  |
|  |  |  |  |  |  |  |

**Supplementary Table 1**. Randomized Clinical Trials (RCTs) quality evaluation. **Y** yes, **nr** not reported.


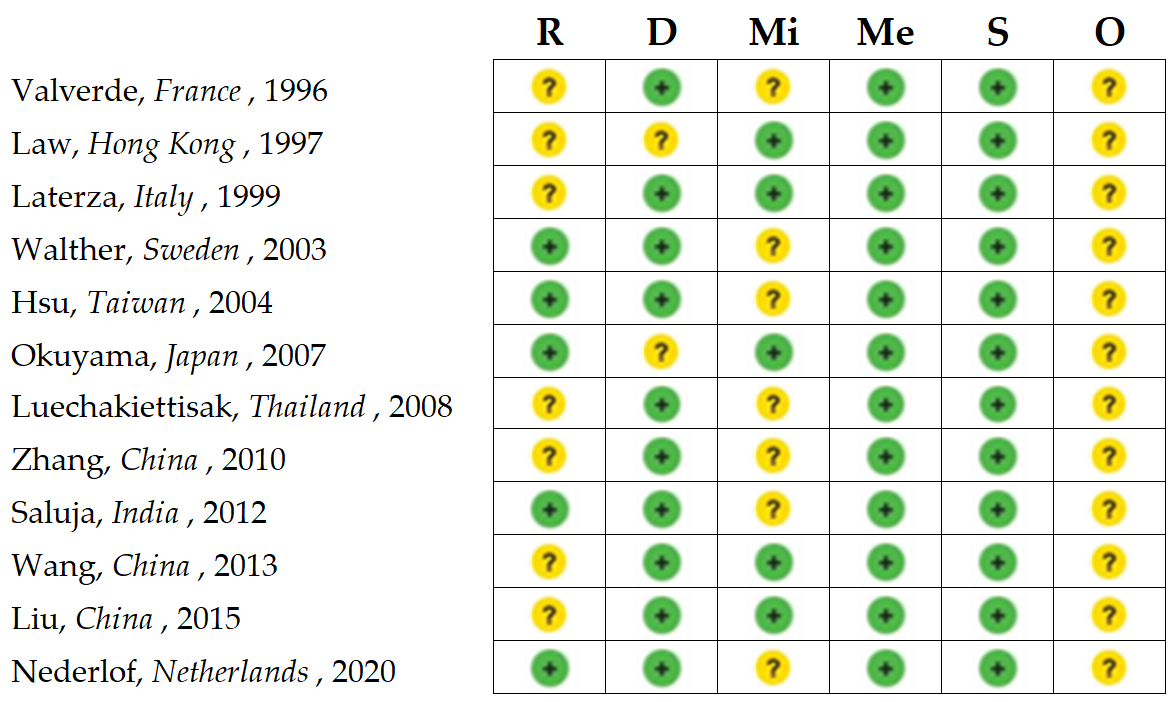


**Supplementary Fig. 1**. Risk of bias for randomized controlled trials (RCT) assessed with use of the Cochrane risk-of-bias tool 2. Green circle: low risk of bias; red circle: high risk of bias; yellow circle: unclear risk of bias. Bias arising from the randomization process (**R**); bias due to deviations from intended interventions (**D**); bias due to missing outcome data (**Mi**); bias in measurement of the outcome (**Me**); bias in selection of the reported result (**S**); overall risk of bias (**O**).

| **Author, country, year** | **Anastomosis specifications** | **Type of suture/** |  |  |  |
| --- | --- | --- | --- | --- | --- |
| **Stapler** | **Anastomosis location T-C** | **Anastomosis type (E-E, E-S, S-S)** | **Surgical procedure (Open-Hybrid-TMI)** |  |  |
| Valverde, France, 1996 ^20^ | HS - One layer | A-NA 71-3 | 50-24 | 14-60-0 | 74-0-0 |
|  | ST - Circular | nr | 57-21 | 4-74-0 | 78-0-0 |
| Law, Hong Kong, 1997 ^21^ | HS- One layer | A, MF | 61-0 | nr | 61-0-0 |
|  | ST - Circular | 18 25mm/30 28mm/ 4 29mm/ 51 31mm/ 4 33mm | 61-0 | 0-61-0 | 61-0-0 |
| Laterza, Italy, 1999 ^22^ | HS - Two layers | A, MF | 0-21 | nr | 21-0-0 |
|  | ST - Circular | 12 21mm/8 25mm | 0-20 | 0-20-0 | 20-0-0 |
| Walther, Sweden, 2003 ^23^ | HS - nr | A, MF | 0-41 | 41-0-0 | 41-0-0 |
|  | ST - Circular | 24 25mm/ 14 28mm/ 4 31mm | 42-0 | 0-42-0 | 42-0-0 |
| Hsu, Taiwan, 2004 ^24^ | HS - Two layers | A, MF; NA, PF | 0-32 | 0-32-0 | 32-0-0 |
|  | ST - Circular | 31 21mm | 0-31 | 0-31-0 | 31-0-0 |
| Okuyama, Japan, 2007 ^25^ | HS - Two layers | A, MF; NA, PF | 0-18 | nr | 18-0-0 |
|  | ST - Circular | 14 25mm | 14-0 | 0-14-0 | 14-0-0 |
| Luechakiettisak, Thailand, 2008 ^26^ | HS - One layer | A, PF | 59-0 | nr | 59-0-0 |
|  | ST - Circular | 40 25mm/ 18 31mm | 58-0 | nr | 58-0-0 |
| Zhang, China, 2010 ^27^ | HS - Two layers | A, PF; NA, PF | 244-0 | 244-0-0 | 244-0-0 |
|  | ST - Circular | 272 25mm | 272-0 | 0-272-0 | 272-0-0 |
| Saluja, India, 2012 ^28^ | HS - Two layers | NA, PF | 0-87 | 0-87-0 | 87-0-0 |
|  | ST - Linear | nr | 0-87 | 0-0-87* | 87-0-0 |
| Wang, China, 2013 ^29^ | HS - One layer | nr | 44-8 | 52-0-0 | 52-0-0 |
|  | ST - Circular | 47 25mm | 70-22 | 0-47-45 | 92-0-0 |
| Liu, China, 2015 ^30^ | HS - One layer | A, MF | 113-354 | 232-0-0 | 237-0-0 |
|  | ST - Circular | 58 25mm/171 29mm/ 6 33mm |  | 0-235-0 | 241-0-0 |
| Nederlof, Netherlands, 2020 ^31^ | HS - One layer | A, MF | 0-44 | 44-0-0 | 44-0-0 |
|  | ST - Linear | nr | 0-49 | 0-0-49^#^ | 49-0-0 |

**Supplementary Table 2** Technical anastomosis data. HS Hand-sewn anastomosis. ST Stapled anastomosis. MF=mono-filament, PF=pluri-filament, NA=non-absorbable, A=absorbable, T=Toracic, C=Cervical, E-E= end to end, E-S= end to side, S-S= side to side, TMI=Totally minimally invasive *Orringer ^#^Collard
