# Supplementary material for: Stapled vs. hand-sewn anastomosis during esophagectomy: a randomized trials systematic review and meta-analysis
Source: Updates Surg. 2025 Nov 21;78(1):95–106. doi: 10.1007/s13304-025-02464-y (PMC12909439; doi:10.1007/s13304-025-02464-y)
Supplement: Supplementary file 2 — Supplementary Material 2 [file 13304_2025_2464_MOESM2_ESM.docx]

**Appendix 1**

**PubMed**

(("Esophagectomy"[Mesh] OR Esophagectomy[tiab] OR Esophageal[tiab] OR Esophagus[tiab])

AND

("Esophageal Neoplasms"[Mesh] OR Cancer[tiab])

AND

(Hand-sewn[tiab] OR Stapler[tiab])

AND

("Surgical Anastomosis"[Mesh] OR Anastomosis[tiab] OR Anastomotic[tiab])

AND

Leak[tiab])

**Scopus**

(TITLE-ABS-KEY (Esophagectomy OR Esophageal OR Esophagus))

AND

(TITLE-ABS-KEY (Cancer))

AND

(TITLE-ABS-KEY (Hand-sewn OR Stapler))

AND

(TITLE-ABS-KEY (Anastomosis OR Anastomotic))

AND

(TITLE-ABS-KEY (Leak))

**Web of Science**

TS=(Esophagectomy OR Esophageal OR Esophagus)

AND

TS=(Cancer)

AND

TS=(Hand-sewn OR Stapler)

AND

TS=(Anastomosis OR Anastomotic)

AND

TS=(Leak)

**Cochrane Central Library**

(Esophagectomy OR Esophageal OR Esophagus)

AND

Cancer

AND

("Hand-sewn" OR Stapler)

AND

anastomos*

AND

(Leak OR Leakage)

**Google scholar**

(Esophagectomy OR Esophageal OR Esophagus) AND Cancer AND ("Hand-sewn" OR Stapler) AND (Anastomosis OR Anastomotic) AND (Leak OR Leakage)

**ClinicalTrials.gov**

Esophagectomy AND Cancer AND ("Hand-sewn" OR Stapler) AND (Anastomosis OR Anastomotic) AND (Leak OR Leakage)
